# Supplementary material for: Late quaternary biotic homogenization of North American mammalian faunas
Source: Nat Commun. 2022 Jul 8;13:3940. doi: 10.1038/s41467-022-31595-8 (PMC9270452; doi:10.1038/s41467-022-31595-8)
Supplement: Supplementary file 1 — Supplementary Information [file 41467_2022_31595_MOESM1_ESM.pdf]

Supplementary Information: Late Quaternary Biotic Homogenization of North American Mammalian Faunas.

A Starting communities

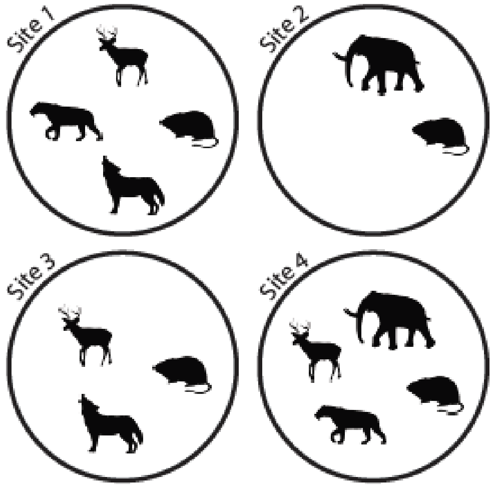

	Site1	Site2	Site3	Site4
Mammuthus	0	1	0	1
Smilodon	1	0	0	1
Peromyscus	1	1	1	1
Canis	1	0	1	0
Odocolleus	1	0	1	1

Mean taxonomic similarity = 0.45

B Extinction of narrow-ranging taxa

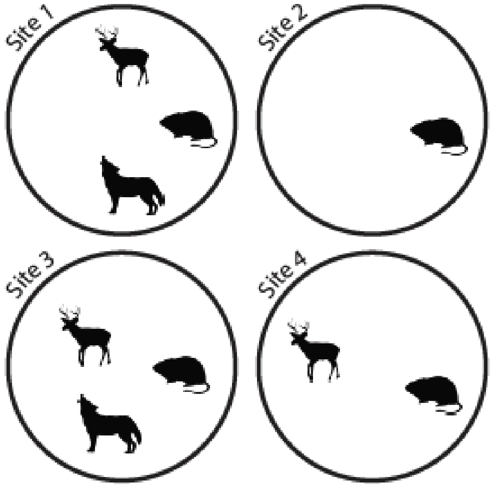

	Site1	Site2	Site3	Site4
Peromyscus	1	1	1	1
Canis	1	0	1	0
Odocolleus	1	0	1	1

Mean taxonomic similarity = 0.58

C Range expansion among survivors

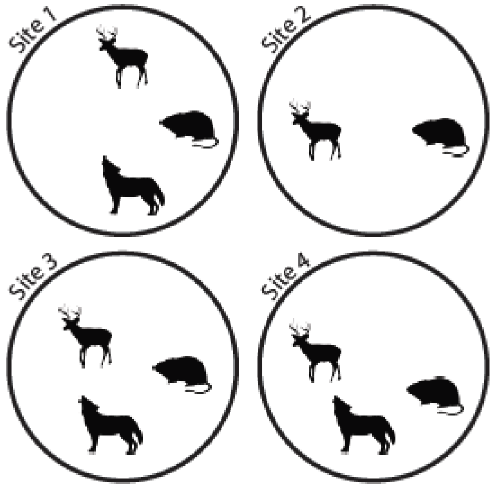

	Site1	Site2	Site3	Site4
Peromyscus	1	1	1	1
Canis	1	0	1	1
Odocolleus	1	1	1	1

Mean taxonomic similarity = 0.83

D Extinction of wide-ranging taxa

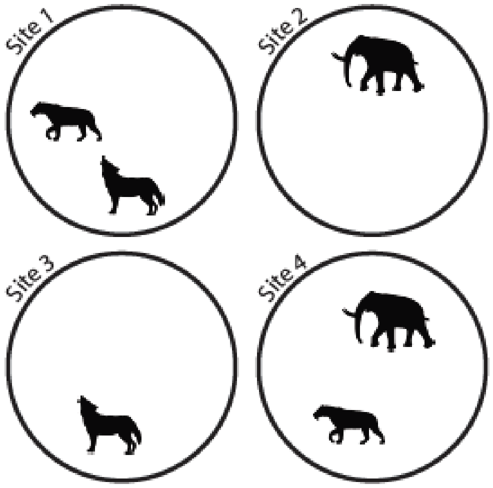

	Site1	Site2	Site3	Site4
Mammuthus	0	1	0	1
Smilodon	1	0	0	1
Canis	1	0	1	0

Mean taxonomic similarity = 0.22

SUPPLEMENTARY FIGURE 1. Hypothetical scenarios showing changes in mean Jaccard similarity under different scenarios of extinction and range size change. Each circle represents a hypothetical site where fossils have been collected; B, C, and D can be compared with starting community in A. Each table represents a hypothetical site by genus occurrence matrix (1 represents presence and 0 represents absence). Animal silhouettes are distributed under Public Domain Dedication 1.0 license.

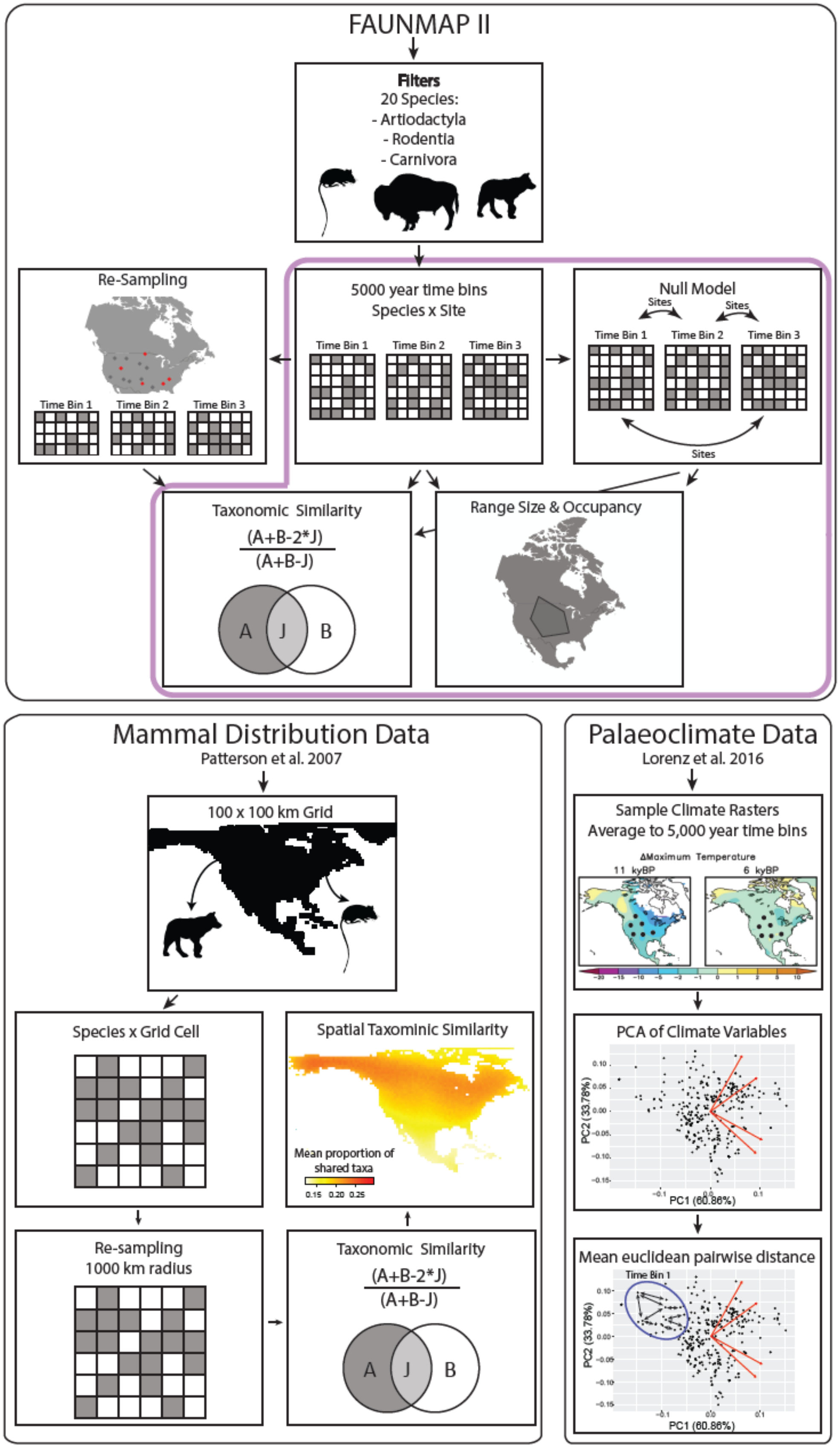
FAUNMAP II
Filters
20 Species:
- Artiodactyla
- Rodentia
- Carnivora
Re-Sampling
Time Bin 1
Time Bin 2
Time Bin 3
5000 year time bins
Species x Site
Null Model
Sites
Taxonomic Similarity
(A+B-2*J)
(A+B-J)
A
J
B
Range Size & Occupancy
Mammal Distribution Data
Patterson et al. 2007
100 x 100 km Grid
Species x Grid Cell
Spatial Taxominic Similarity
Mean proportion of shared taxa
Re-sampling
1000 km radius
Palaeoclimate Data
Lorenz et al. 2016
Sample Climate Rasters
Average to 5,000 year time bins
ΔMaximum Temperature
11 kyBP
6 kyBP
PCA of Climate Variables
PC1 (60.86%)
PC2 (33.78%)
Mean euclidean pairwise distance

SUPPLEMENTARY FIGURE 2. Visual summary of methods. Methods outlined in purple were repeated for mammals larger than 1 kg and 5 kg, without the mammal megafauna, excluding sites in the Rockies, and excluding sites north of the Canadian border. Silhouette credits from phylopic.org: Nina Skinner (*Peromyscus*) distributed under the Creative Commons Attribution 3.0 Unported license and Lukasiniho (*Bison*) distributed under Creative Commons Attribution-NonCommercial-ShareAlike 3.0 Unported license. The wolf silhouette is distributed under the Public Domain Dedication 1.0 license. Maximum temperature maps are modified from (68). Remaining images were created by D. Fraser.

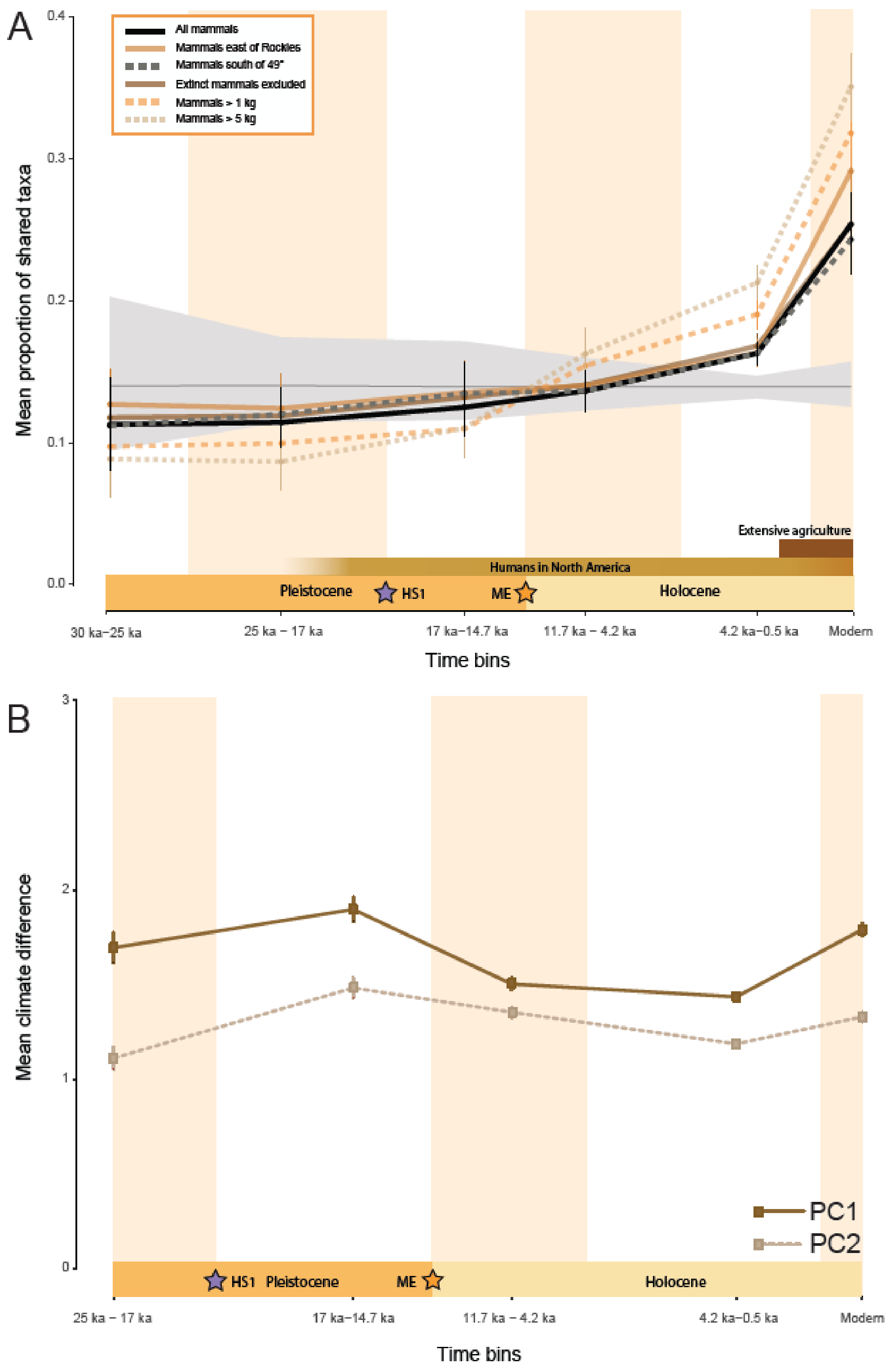

SUPPLEMENTARY FIGURE 3. Mammal mean proportion of shared taxa and mean climate difference calculated using different bin date cut offs from those used in our primary analyses

and shown in Figs. 1, 3, and 4. (A) Mammal assemblages undergo biotic homogenization during the Holocene (sample sizes in Source Data). Mammals larger than 1 kg commence homogenizing during the 17,000-11,700 ybp time bin, but the onset of homogenization is delayed until after 11,700 ybp for assemblages including all mammals. Mammals larger than 1 kg are more homogenous than null expectations by the 11,700-4,200 ybp time bin while assemblages of all mammals are more homogenous than null expectations by the 4,200-500 ybp time bin. Change in mean taxonomic similarity (Jaccard similarity index) among sites +/- the standard error of the mean. Gray ribbon shows the mean of the null model runs (sites shuffled among time bins) with 95% confidence intervals. Dates of the mammal sites are based on calibrated radiocarbon dates (See Material and Methods). (B) Mean climate difference does not decrease during the intervals for which we observe biotic homogenization (sample sizes in Source Data). Change in climate turnover (mean climate difference) +/- the standard error of the mean. Climate estimates are based on de-biased and downscaled earth system model (ESM) climate simulations from recent and paleoclimate models at 0.5 degree resolution (1). The orange star indicates the extinction of the mammal megafauna in North America (ME). The purple star indicates the beginning of Heinrich Stadial 1 (HS1). The modern time bin (1980's – 2010's) is portrayed as larger to enhance readability.

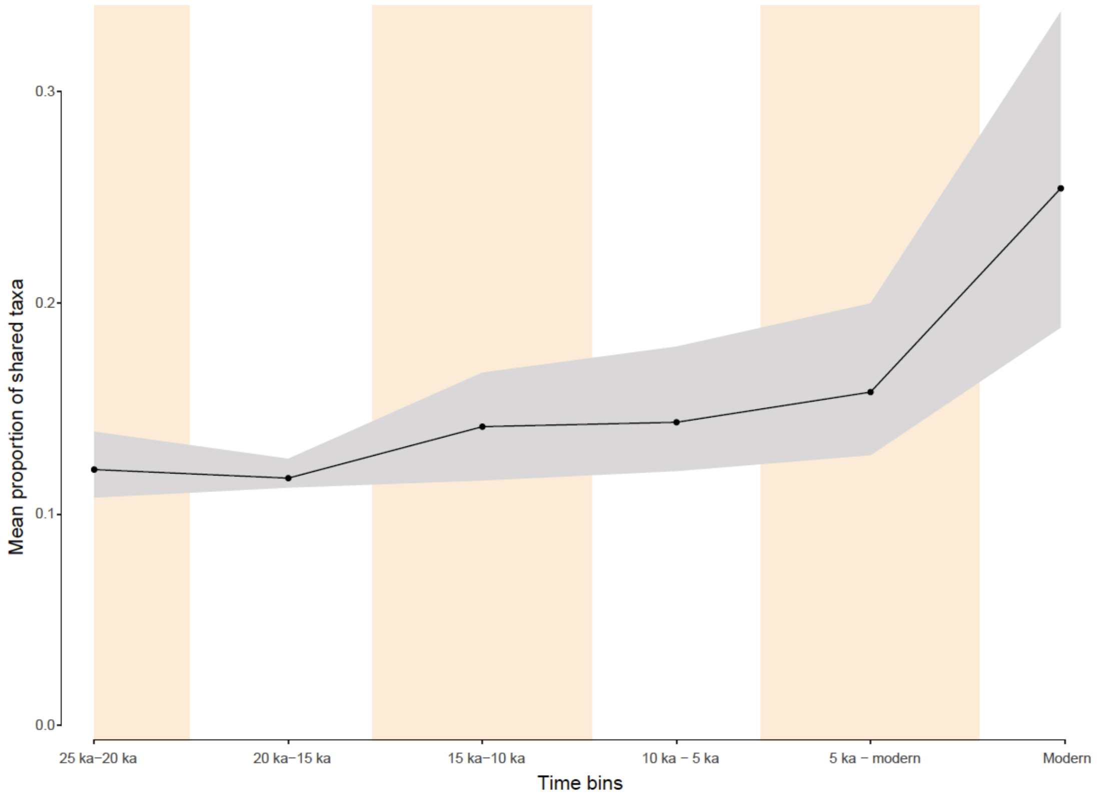

SUPPLEMENTARY FIGURE 4. Biotic homogenization is apparent when using a re-sampling approach (*n* = 366 sites, 365 unique species). Mean Jaccard taxonomic similarity for mammals with 95% confidence intervals when subsampling is limited to 15 sites in each time interval.

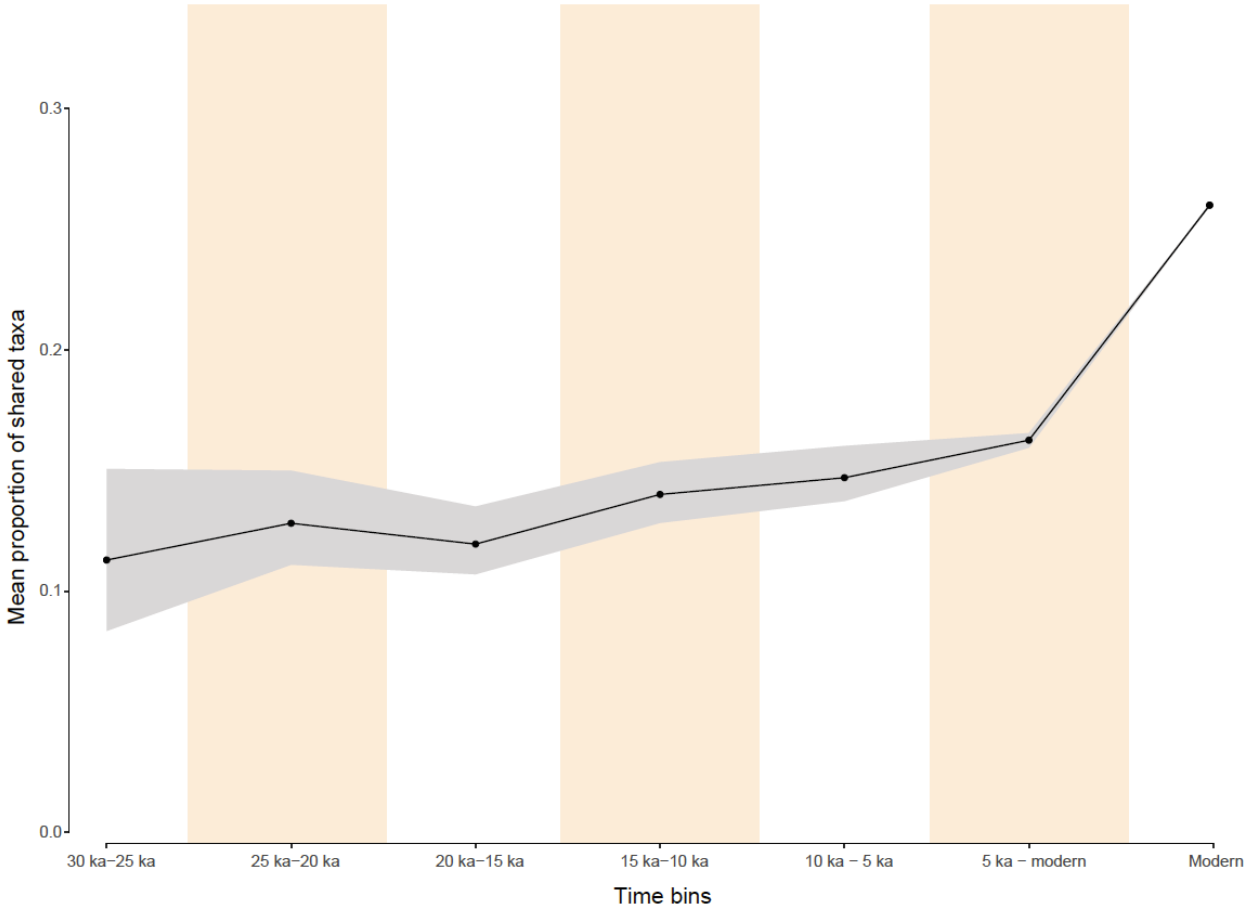

SUPPLEMENTARY FIGURE 5. Biotic homogenization is apparent for North American mammals when accounting for potential bulk radiocarbon dating errors (n = 366 sites, 365 unique species)**.** Mean Jaccard taxonomic similarity with 95% confidence intervals for each 5000 year time interval when radiocarbon dating errors are drawn randomly from a normal distribution with a mean of zero and standard deviation of 2,000 years.

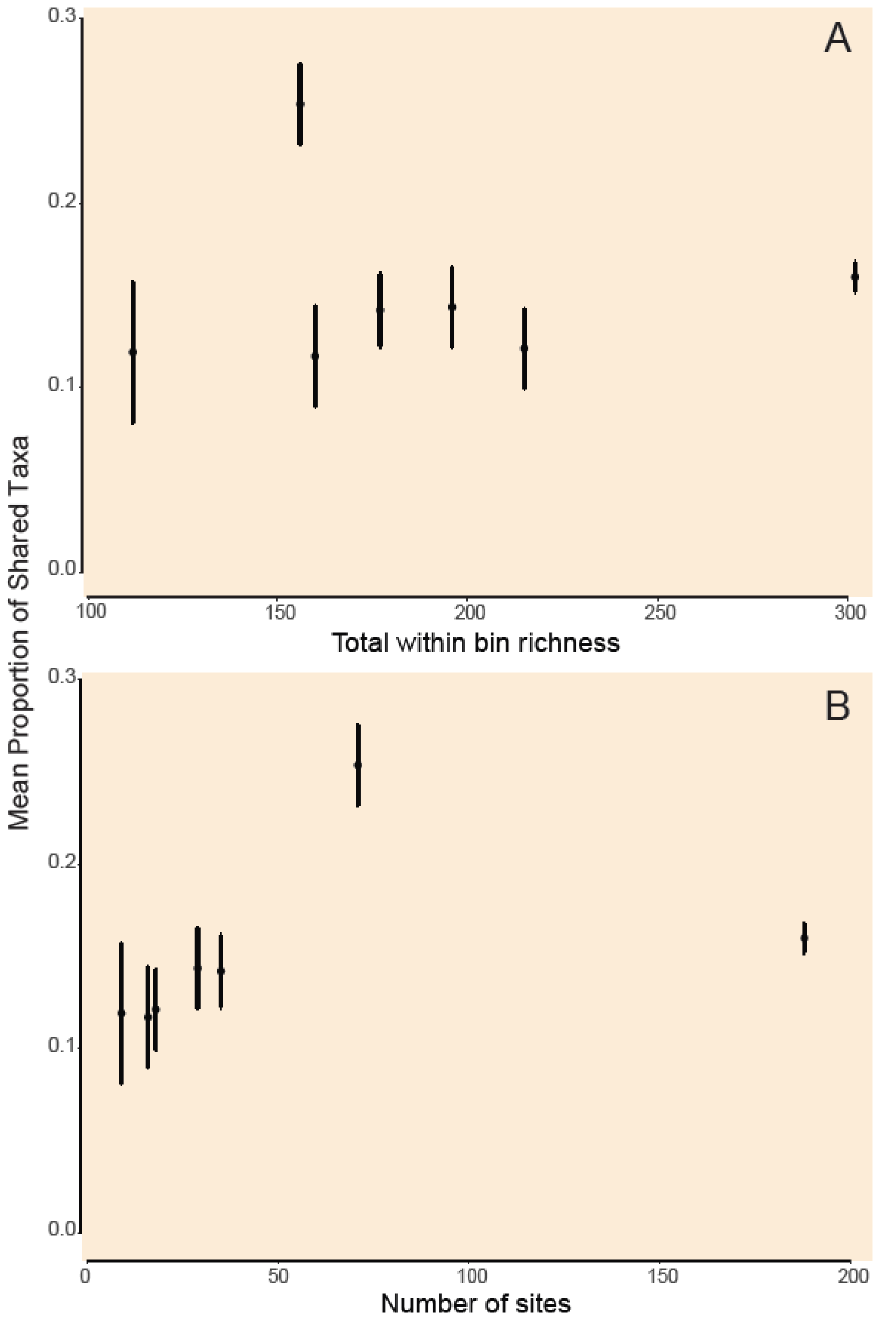

SUPPLEMENTARY FIGURE 6. There is no correlation between γ diversity and the mean proportion of shared taxa during the late Pleistocene and Holocene (*n* = 366 sites, 365 unique species). (A) The relationship between γ diversity and mean Jaccard taxonomic similarity for each 5,000 year time interval. (B) Residuals from a regression of γ diversity against mean Jaccard similarity through time. Vertical lines are the mean +/- the standard error of the mean.

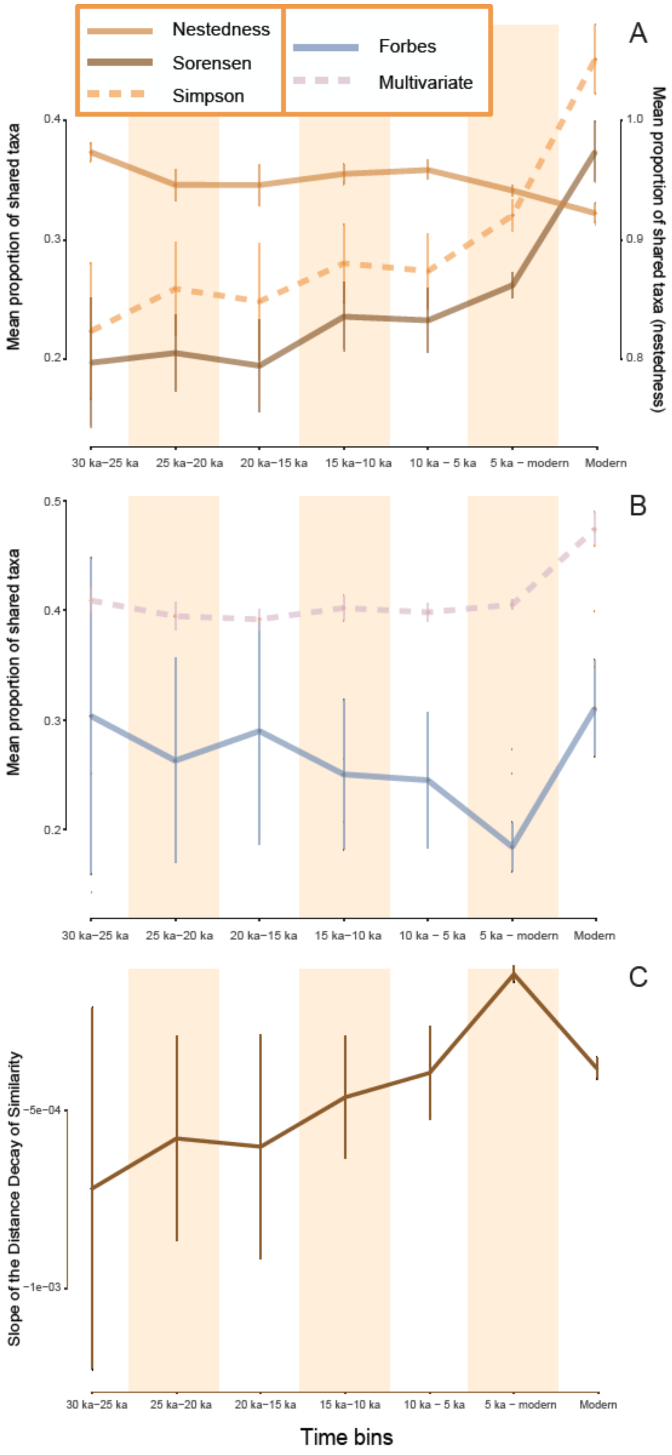

SUPPLEMENTARY FIGURE 7. Biotic homogenization remains apparent when using different measures of taxonomic similarity (*n* = 366 sites, 365 unique species). Change in mean taxonomic

similarity quantified using (A) Baselga's partitioning of the turnover and nestedness components β diversity, (B) other proposed methods for quantifying taxonomic similarity among sites, and (C) distance decay of similarity, which is the slope of a line fit to a regression of taxonomic similarity against distance. The slight downturn in the last time bin reflects the inclusion of Alaskan sites. All vertical lines represent the mean +/- the standard error of the mean.

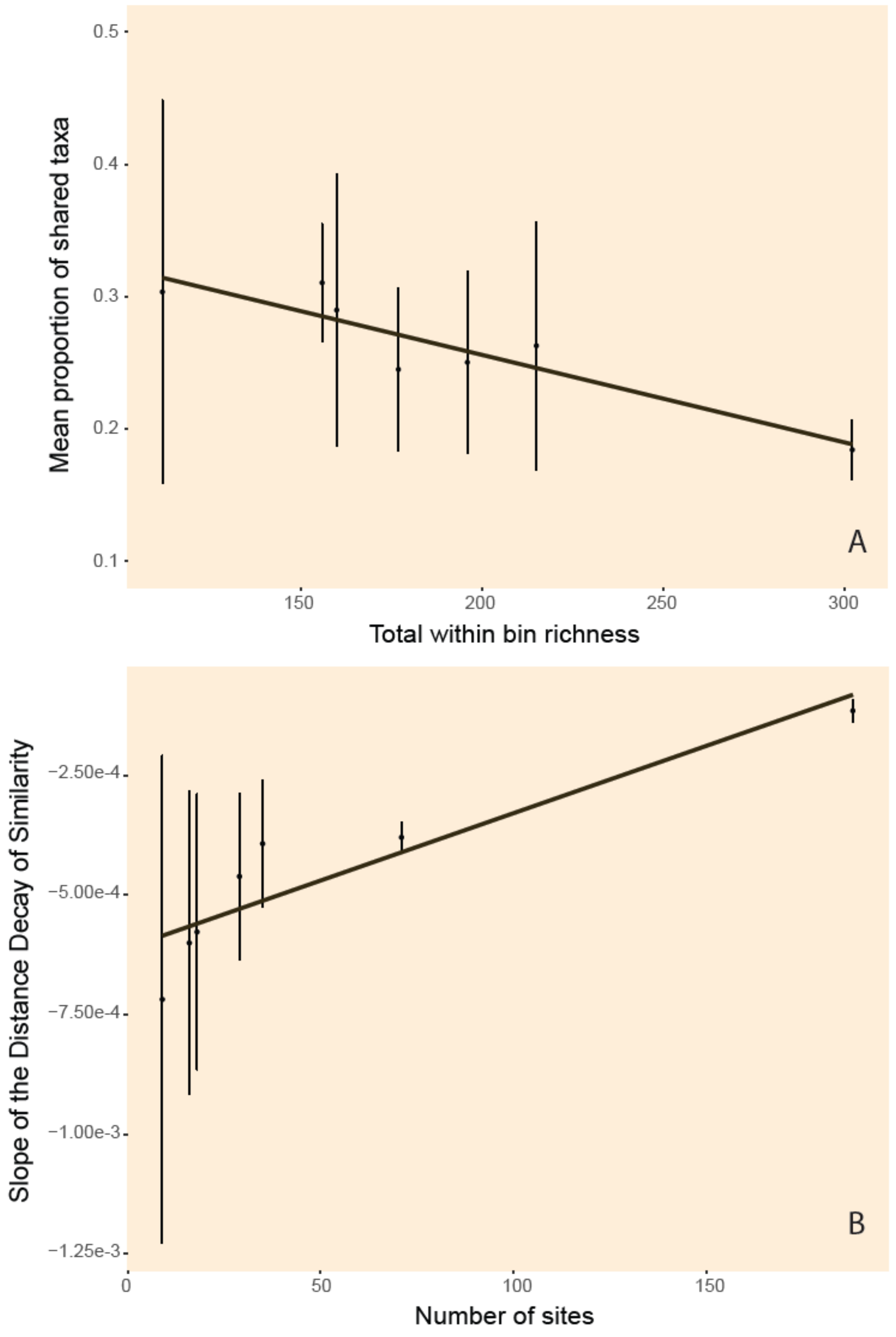

SUPPLEMENTARY FIGURE 8. There are significant correlations between (a) γ diversity for each 5,000 year time interval and the mean proportion of shared taxa as calculated using Forbes similarity and (b) the number of sites for each 5,000 year time interval and the slope of the

Distance Decay of Similarity during the late Pleistocene and Holocene (*n* = 366 sites, 365 unique species). Vertical lines are the mean +/- the standard error of the mean.

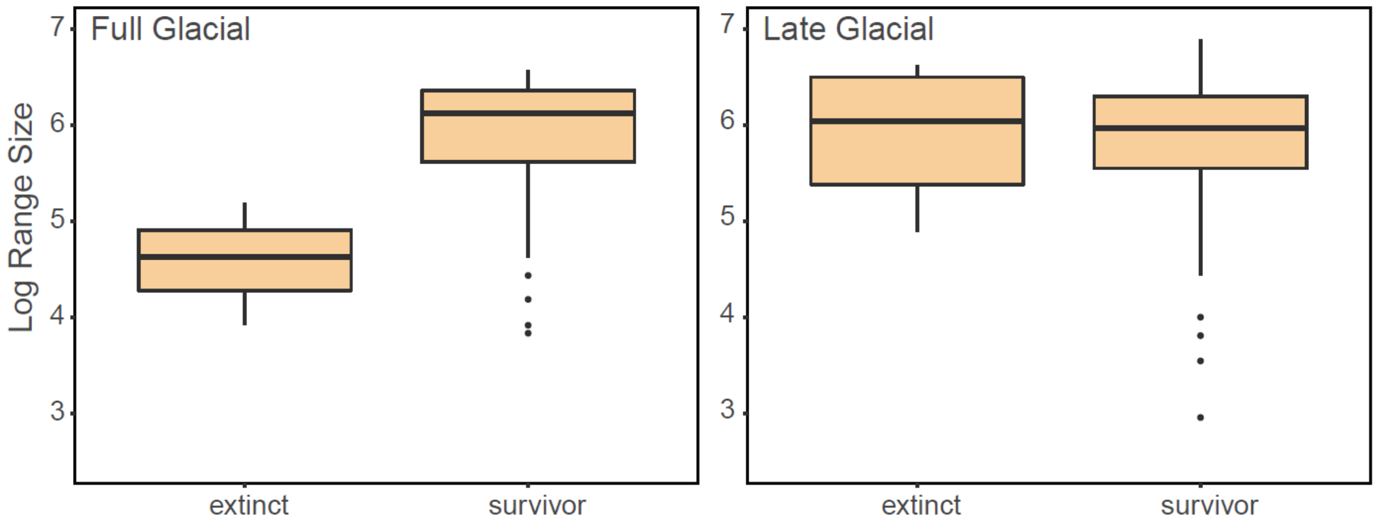

SUPPLEMENTARY FIGURE 9. Mean geographic range size during the full glacial and de-glaciation periods is smaller for species that go extinct at the end of the Pleistocene (*n* = 140 unique species). This should have favored biotic homogenization, but exclusion of extinct fauna from our analyses does not change the pattern we observe. Thick black horizontal lines are the median. The lower and upper hinges are the first and third quartiles. The upper and lower whiskers represent the largest and smaller values no furthe than 1.5 times the inter-quartile range. Dots represent outliers.

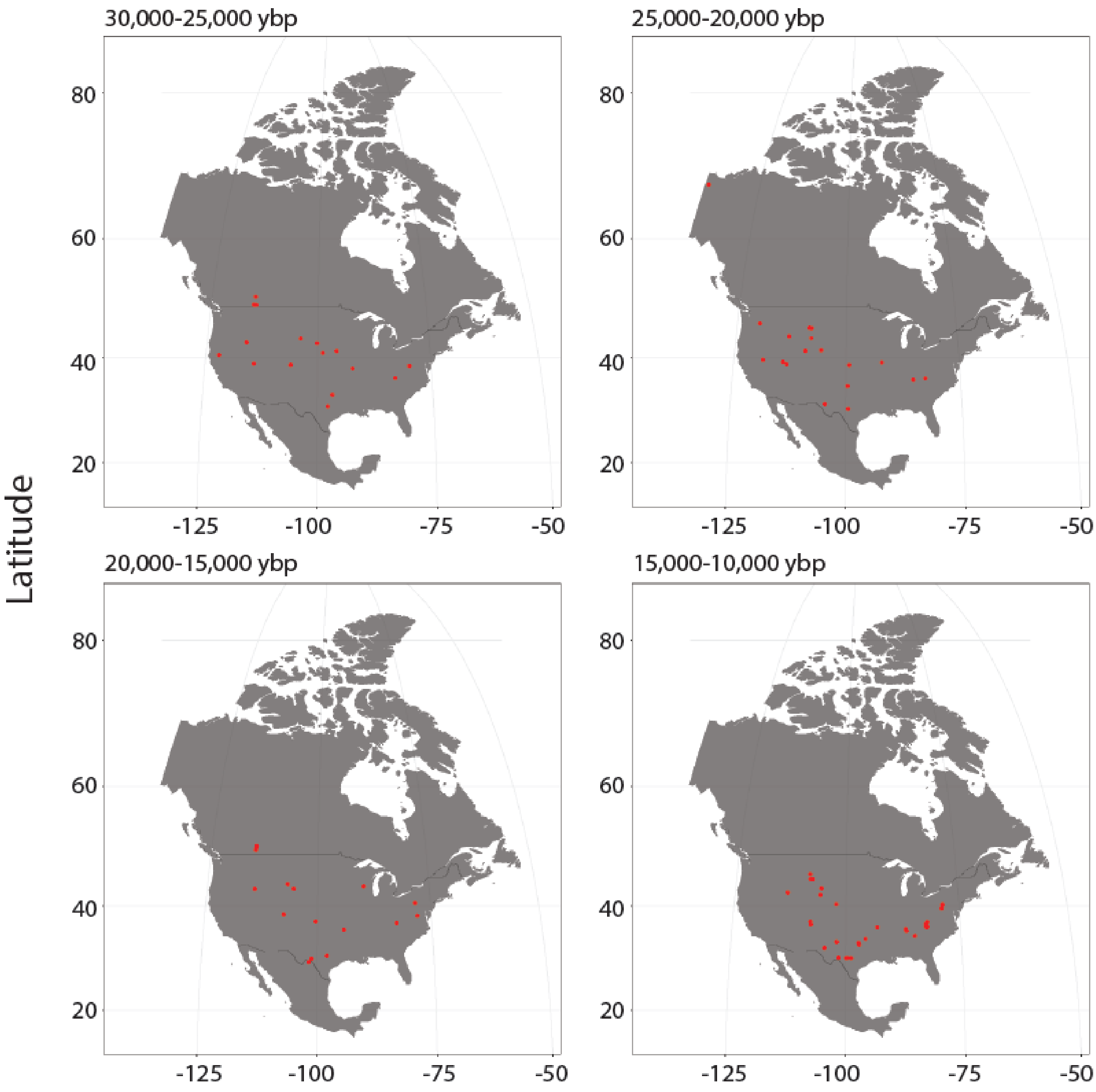
30,000-25,000 ybp
25,000-20,000 ybp
20,000-15,000 ybp
15,000-10,000 ybp
Latitude
80
60
40
20
-125
-100
-75
-50

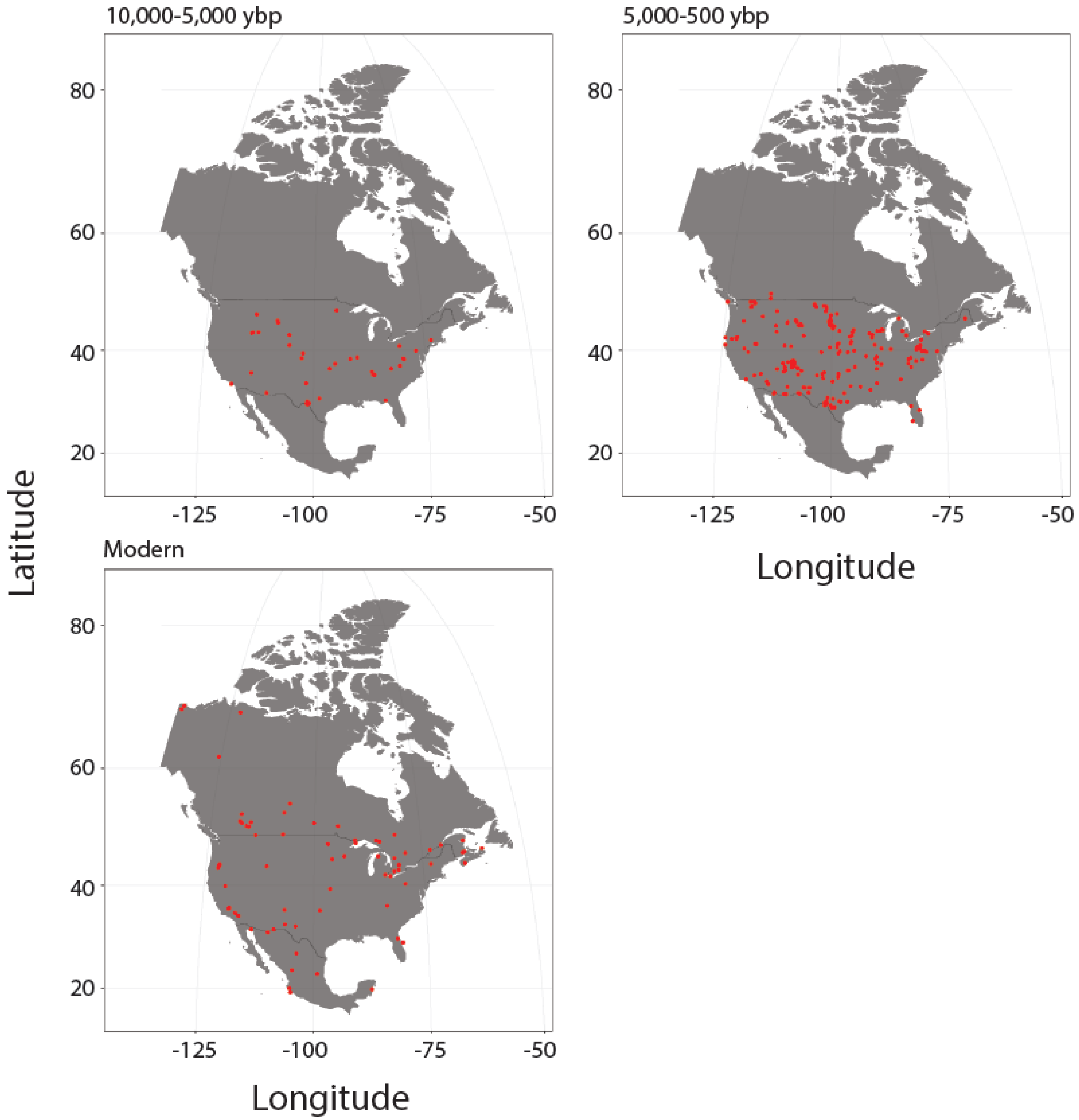

SUPPLEMENTARY FIGURE 10. Distributions of mammal localities for all time bins. Sample sizes in Source Data. Maps were created by D. Fraser and A. Villaseñor.

1. Lorenz DJ, Nieto-Lugilde D, Blois JL, Fitzpatrick MC, & Williams JWJSd (2016) Downscaled and debiased climate simulations for North America from 21,000 years ago to 2100AD. 3:160048.
