# Supplementary material for: Late quaternary biotic homogenization of North American mammalian faunas
Source: Nat Commun. 2022 Jul 8;13:3940. doi: 10.1038/s41467-022-31595-8 (PMC9270452; doi:10.1038/s41467-022-31595-8)
Supplement: Supplementary file 3 — Reporting Summary [file 41467_2022_31595_MOESM3_ESM.pdf]

nature portfolio

Corresponding author(s): Fraser, Danielle

Last updated by author(s): May 16, 2022

Reporting Summary

Nature Portfolio wishes to improve the reproducibility of the work that we publish. This form provides structure for consistency and transparency in reporting. For further information on Nature Portfolio policies, see our Editorial Policies and the Editorial Policy Checklist.

Statistics

For all statistical analyses, confirm that the following items are present in the figure legend, table legend, main text, or Methods section.

n/a	Confirmed	
☐	☒	The exact sample size (*n*) for each experimental group/condition, given as a discrete number and unit of measurement
☐	☒	A statement on whether measurements were taken from distinct samples or whether the same sample was measured repeatedly
☒	☐	The statistical test(s) used AND whether they are one- or two-sided<br>*Only common tests should be described solely by name; describe more complex techniques in the Methods section.*
☐	☒	A description of all covariates tested
☒	☐	A description of any assumptions or corrections, such as tests of normality and adjustment for multiple comparisons
☐	☒	A full description of the statistical parameters including central tendency (e.g. means) or other basic estimates (e.g. regression coefficient) AND variation (e.g. standard deviation) or associated estimates of uncertainty (e.g. confidence intervals)
☒	☐	For null hypothesis testing, the test statistic (e.g. *F*, *t*, *r*) with confidence intervals, effect sizes, degrees of freedom and *P* value noted<br>*Give P values as exact values whenever suitable.*
☒	☐	For Bayesian analysis, information on the choice of priors and Markov chain Monte Carlo settings
☒	☐	For hierarchical and complex designs, identification of the appropriate level for tests and full reporting of outcomes
☐	☒	Estimates of effect sizes (e.g. Cohen's *d*, Pearson's *r*), indicating how they were calculated

Our web collection on statistics for biologists contains articles on many of the points above.

Software and code

Policy information about availability of computer code

Data collection	New data were not collected for the present study.
Data analysis	All analyses were performed in the R programming language. All R code used during the current study are available in the Github repository [https://doi.org/10.5281/zenodo.6518845]. R packages used include Bchron v. 4.7.5, betapart v. 1.5.4, vegan v. 2.5.7, fossil v. 0.4.0, glm2 v. 1.2.1, sp v. 1.4.5, raster v. 3.4.10, maptools v. 1.1.1, adehabitatHR v. 0.4.19, and ggplot2 v. 3.3.5. Their use is shown in the available R code.

For manuscripts utilizing custom algorithms or software that are central to the research but not yet described in published literature, software must be made available to editors and reviewers. We strongly encourage code deposition in a community repository (e.g. GitHub). See the Nature Portfolio guidelines for submitting code & software for further information.

Data

Policy information about availability of data

All manuscripts must include a data availability statement. This statement should provide the following information, where applicable:

- Accession codes, unique identifiers, or web links for publicly available datasets
- A description of any restrictions on data availability
- For clinical datasets or third party data, please ensure that the statement adheres to our policy

Pleistocene and Holocene mammal data were collected from the published Faunmap 2.0 database (https://ucmp.berkeley.edu/faunmap/). Data from individual fossil and archaeological sites was not collected by the authors of the present study. Faunmap was assembled from published occurrence data (described here: https://ucmp.berkeley.edu/faunmap/). Modern mammal occurrence data were collected from the following sources, Brown & Nicoletto (1991) and Lyons & Smith (2013). Brown & Nicoletto (1991) and Lyons & Smith (2013) compiled mammal occurrence data various sources, including within national parks, as described therein. Software used in data collection included an internet browser (Google Chrome) and Microsoft excel. The data used in this study have been deposited in the

Github repository [https://doi.org/10.5281/zenodo.6518845].

Field-specific reporting

Please select the one below that is the best fit for your research. If you are not sure, read the appropriate sections before making your selection.

[ ] Life sciences   [ ] Behavioural & social sciences   [x] Ecological, evolutionary & environmental sciences

For a reference copy of the document with all sections, see nature.com/documents/nr-reporting-summary-flat.pdf

Ecological, evolutionary & environmental sciences study design

All studies must disclose on these points even when the disclosure is negative.

Study description	Using the occurrences of Pleistocene and Holocene mammals in North America, we tested for increases in mean community similarity (i.e., biotic homogenization) over the past 30,000 years.
Research sample	For the Pleistocene and early to mid Holocene, we used occurrences of mammals at fossil sites (e.g., caves, holes in the ground, rock outcrops) from Faunmap 2.0 (https://ucmp.berkeley.edu/faunmap/), vetted to include 20 or more species from Artiodactyla, Carnivora, and Rodentia. We chose sites with a minimum of 20 species for consistency with Brown & Nicoletto (1991) and to eliminate poorly sampled sites. The dataset of modern mammal occurrences were taken from a dataset compiled by S.K. Lyons from a variety of literature and web sources (Lyons & Smith, 2013). Our total sample includes 8,831 mammal occurrences, 365 mammal species, and 366 localities. Both vetted and cleaned datasets are now available on github [https://doi.org/10.5281/zenodo.6518845] in the form of a species by site occurrence matrix.
Sampling strategy	Sites were included in our analysis only if at least 20 species of mammal were recorded and those 20 species included representatives from the Artiodactyla, Carnivora, and Rodentia, thus representing the clades that comprise the majority of non-volent mammal diversity. Our procedure ensures that poorly sampled sites are excluded from the analysis. We also chose sites with a minimum of 20 species for consistency with Brown & Nicoletto (1991).
Data collection	No new data were collected for the present study.
Timing and spatial scale	No new data were collected for the present study. We included sites that were dated either by direct dating (i.e., radiocarbon dating) or biostratigraphy to the late Pleistocene, Holocene (>= 500 years of age). Modern mammal occurrences are observational, dating to the 1970's-2,000's. All sites were restricted to North America. Maps of the spatial coverage of sites are included in the supplementary information.
Data exclusions	We excluded sites that did not include a minimum of 20 species belonging to the Artiodactyla, Carnivora, and Rodentia.
Reproducibility	All data and code are available on Github (https://github.com/danielleleefraser/biotic_homogenization).
Randomization	Sites were divided among time bins based on either direct dating i(.e., radiocarbon dating) or biostratigraphy. No randomization was used except during the construction of our null model, which involved shuffling sites (with their entire complement of species) among time bins while maintaining the total number of sites per time bin. The null model was used to calculate effect sizes.
Blinding	No new data were collected for the present study, thus a blinding procedure was not required or possible.

Did the study involve field work? [ ] Yes [x] No

Reporting for specific materials, systems and methods

We require information from authors about some types of materials, experimental systems and methods used in many studies. Here, indicate whether each material, system or method listed is relevant to your study. If you are not sure if a list item applies to your research, read the appropriate section before selecting a response.

Materials & experimental systems

n/a	Involved in the study	
[x]	[ ]	Antibodies
[x]	[ ]	Eukaryotic cell lines
[ ]	[x]	Palaeontology and archaeology
[x]	[ ]	Animals and other organisms
[x]	[ ]	Human research participants
[x]	[ ]	Clinical data
[x]	[ ]	Dual use research of concern

Methods

n/a	Involved in the study	
[x]	[ ]	ChIP-seq
[x]	[ ]	Flow cytometry
[x]	[ ]	MRI-based neuroimaging

Palaeontology and Archaeology

Specimen provenance	The fossil occurrence data were downloaded from Faunmap 2.0, which is a compendium of published fossil mammal occurrences during the Pleistocene through Holocene (>= 500 years) in North America. The present study did not involve the direct examination of fossil or archaeological material.
Specimen deposition	The specimens referenced in the Faunmap 2.0 database are deposited at natural history museums throughout the USA and Canada. The present study did not involve the direct examination of fossil or archaeological material.
Dating methods	No new dates are provided. Radiocarbon dates were calibrated using using the IntCal13 calibration curve and the R package Bchron.

[x] Tick this box to confirm that the raw and calibrated dates are available in the paper or in Supplementary Information.

Ethics oversight	No ethical approval was required.

Note that full information on the approval of the study protocol must also be provided in the manuscript.
